# A Secure Enhanced Non-Cooperative Cognitive Division Multiple Access for Vehicle-to-Vehicle Communication

**DOI:** 10.3390/s20041000

**Published:** 2020-02-13

**Authors:** Mohammed Abdulhakim Al-Absi, Ahmed Abdulhakim Al-Absi, Hoon Jae Lee

**Affiliations:** 1 Department of Computer Engineering, Graduate School, Dongseo University, 47 Jurye-ro, Sasang-gu, Busan 47011, Korea; mohammed.a.absi@gmail.com; 2 Department of Smart Computing, Kyungdong University 46 4-gil, Bongpo, Gosung, Gangwon-do 24764, Korea; absiahmed@kduniv.ac.kr; 3 Division of Information and Communication Engineering, Dongseo University, 47 Jurye-ro, Sasang-gu, Busan 47011, Korea

**Keywords:** radio propagation, vanet, MAC, vehicle-to-vehicle, line of sight

## Abstract

The growth of the Internet has led to the increasing usage of smart infotainment applications on the vehicular ad-hoc network (VANET). Preserving privacy and security regarding the provision of smart infotainment applications while on the go is most desired. Thus, a secure authentication scheme is required. Many privacy-preserving security schemes have been developed in recent times using cryptography approaches. However, these incur key management and communication overhead. The usage of third-party servers incurs the overhead of key computation, storage and distribution. Post completion of the initialization phase, the message is secured using cryptography and is shared among vehicles. The design of the proposed secure enhanced non-cooperative cognitive division multiple access (S−ENCCMA) aims to eliminate the need for the local message available with the parties to be released for provisioning secure safety-related applications. To overcome the research challenges, this work presents a novel security scheme, namely secure non-cooperative cognitive medium access (S−ENCCMA). The experiment is conducted to evaluate the overhead incurred in provisioning security to ENCCMA. The outcome shows that the overhead incurred by S−ENCCMA over ENCCMA was negligible to provide the real-time security requirements of smart infotainment applications, which is experimentally shown in this paper in terms of throughput, collision and successful packet transmission considering varied environmental models such as cities, highways and rural areas.

## 1. Introduction

**Figure 1:**
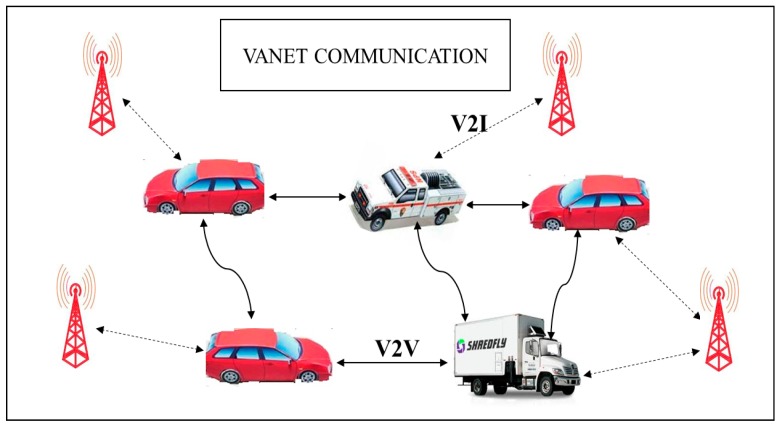
The architecture of vehicular ad-hoc network (VANET) communication.

The vehicular ad-hoc network (VANET) has similar characteristics to the Mobile Ad-Hoc Network (MANET) where the vehicle is mobile in nature and controlled by road topologies [1]. The objective of VANET is to provide the driver and user with a safe and reliable communication environment. The communication in VANET can be broadly classified into a vehicle to vehicle (V2V), vehicle to infrastructure or roadside unit (RSU) (V2I) and V2X, which is a combination of both V2I and V2V. The basic architecture of VANET communication is shown in Figure 1. Each vehicle is embedded with an onboard unit (OBU) that has communicational and computational capabilities [2].

As per the standard stated by Dedicated Short Range Communication (DSRC), a vehicle broadcasts safety-related message or beacons [3]. These messages possess information such as the vehicle direction, location, speed and other traffic-related information such as accidents and congestion. This aids in assisting vehicle drivers or users to take its contextual corresponding decisions in order to avoid an accident and congested routes. However, preserving the privacy of such information is considered a critical element, since it may breach the privacy concern of the user. For example, the starting and ending positions of a particular vehicle may generally be the information of the office and home addresses or vice versa of a user. There are also some security concerns, i.e., there is a risk if an intruder beacons a false message to gain unfair access, which may lead to congestion or in the worst case may lead to an accident or loss of life. In order to take part in VANET communication, a valid user must be authenticated. However, authenticating VANET users in such an environment is challenging, since authentication requires some identity-related information such as the vehicle number, driving license and so on. As a result, exposing such information may breach the privacy concern of users. Hence, it is desired to preserve the privacy of such information. In case of any malicious activity detected, the security design should be able to track the malicious user. These requirements make provisioning security and privacy a key challenge in VANET and for achieving an effective security mechanism, these issues need to be addressed [4] and [5].

Authentication is an important necessity in the VANET, as it assures that the actual node sends the messages and therefore that a greater range of attacks can be reduced, which are done by the greedy drivers or the opponent drivers. However, an authentication increases the concern of privacy; a basic scheme for authentication of providing the identity details of the sender along with the message would help with tracking the vehicles. Therefore, as per the application, authentication should be provided as an absolute essential to authenticate that a certain property is being assigned to the sending vehicles. For instance, in location-based services, that a vehicle is in a particular area where it is stated to be is the property that could state it. Message integrity is also important, because this assures that the message is not being altered during the transition and that the messages that the driver receives are not false. In this security-based system, message non-repudiation ensures that the sender cannot deny the message that has already been sent. However, it does not state that any of the drivers can identify the sender; instead, only the specifically authorized drivers should be allowed to identify the authenticated message from the vehicle sending/transferring the message. Entity authentication assures that the message generated by the sender is still in the network, which allows the driver to understand that the message from the sender is delivered in a shorter period of time. Access control is important and necessary for ensuring that the roles and privileges that are authorized are functioning via the nodes accorded to them in the network. Access control and authorization state what each of the nodes in a network can do and what messages they can create. Message confidentiality is required in a system where the communication is done privately by some of the certain nodes. However, not all the nodes can do that. This type of communication can only be done by law enforcement vehicles that are authorized to convey private information among each other, such as identifying the location of a terrorist or criminal. In this system, privacy is used for ensuring that any unauthorized people do not access the information and cannot view the information; the information should not be leaked to any unauthorized person. Any third party should not be able to track the movements of the vehicle, since it could be a violation of personal privacy. Therefore, there should be some sort of privacy or secrecy for the movements of the vehicles and the messages. However, in the cases related to liability, to determine responsibilities, only specified authorities should be allowed to trace the identities of the user. One should not be allowed to know the future and past location of particular vehicles; location privacy is also very important and required. Real-time guarantees are extremely important in a VANET, as many applications that are related to safety depend on the strict time guarantees. This assures that the time-sensitivity of the safety-related applications can be built into many protocols such as avoiding collisions.

The authorities involved in preserving privacy in the V2V semi-honest trust model are not suitable for provisioning high throughput safety and smart infotainment application. To preserve the privacy of user data, in the literature, many researchers have predominantly adopted cryptography techniques. The cryptography technique relies on the keys for encryption and decryptions, where keys are symmetric or asymmetric in nature.

The usage of third-party servers incurs the overhead of key computation, storage, and distribution, which is also known as the initialization phase. Post completion of the initialization phase, the message is secured using cryptography and is shared among vehicles. The design of the proposed secure enhanced non-cooperative cognitive division multiple access (S−ENCCMA) aims to eliminate the need for the local message available with the parties to be released for provisioning secure safety-related applications. A security model is designed using Commutative Rivest–Shamir–Adleman (CRSA), namely S−ENCCMA.

The research work contribution is as follows:

- The message integrity and privacy are preserved using the commutative RSA cryptography technique.
- The S−ENCCMA model preserves privacy in the presence of non-trusted or dishonest authorities.
- Our design incurs much lower overhead in providing security when compared to existing design considering throughput, collision and successful packet transmission performances.

The rest of the paper is organized as follows. An extensive research survey is carried out in Section 2. In Section 3, the proposed S−ENCCMA model is presented. In the penultimate section, an experimental study is carried out. The conclusion and future work are described in the last section.

## 2. Related Work

Recently, many privacy-preserving authentication approaches have been proposed. These approaches can be broadly classified into group-based approaches [6] and pseudonymous-based approaches [7] and [8]. Alongside, there are Mix-zones, RSU-assisted [7], and a silent period [9]. These approaches aim at resolving many securities and privacy-related issues in VANETs, but each approach has its own benefits and limitations. The existing pseudonym-based approaches induce significant delay [10], since it adopts public key infrastructure (PKI) by using a digital signature to authenticate the message. As reported in [10], it requires about 20 ms to verify a signature by an on-board unit considering a 400 MHz processor. This may not be an issue in a sparsely populated region, but in a densely populated region, it may incur serious delay for the message verification process. Another limitation of pseudonym-based approaches is the certificate revocation list (CRL), i.e., a certification authority (CA) generates a set of public-key certificates to a vehicle. Then, the vehicle signs the beacon with its private key and broadcasts the beacons with its corresponding public key certificates. However, in the case of revocation, all certificates pertaining to the revoked user need to be added into CRL. The CRL grows exponentially with respect to a number of revoked vehicles. The on-board unit checks the attached certificate each time the vehicle obtains a beacon message. As a result, it induces the computation and communication overhead of an on-board unit. Pseudonymous-based approaches also suffer from trust-related issues, since it requires the complete trust of RSU and certificate authority. The privacy of the user is compromised if the certificate authority turns malicious or been attacked, since the certificate authority has all the information about the vehicle. Similarly, the RSU is located in open areas, and side-channel attacks may compromise the security and privacy of RSU/users. The group signature-based approaches also have certain limitations. As stated in [11], it incurs significant processing overhead on the onboard unit, since the pairing operation needs to check the association among the identity and the signature. Group management is another issue of group signature-based approaches. That is, the group manager can track its member vehicles, since it possesses the complete knowledge of group members. Therefore, choosing the group manager is a complicated and challenging task. A node can leave or join a group at any instance of time in a dynamic environment and the freshly joined group manager may have all knowledge of its members. To overcome the communication overhead due to the CRL, [12] presented a hierarchy of pseudonyms for semi-trusted multi-authority VANET to preserve the privacy of the user. To communicate among different authority pseudonyms with longer sessions is presented, and to communicate with the vehicle a pseudonym with shorter sessions is presented. However, their model suffers from trust issues pertaining to the certification authority. To address the trust-related issue, [13] presented a hardware-based hybrid security model. In their model, firstly the onboard unit first generates its anonymous encryption key to initialize authentication sessions. Secondly, the trusted authority verifies the anonymity of the users. The trust parameter is evaluated using the behavior of the user. Post evaluation of the trust parameter, a session key is generated for commencing communication among vehicles. Their model adopts bilinear pairing, which aids in minimizing the key management overhead. However, the tamper-proof hardware cannot address all the security requirements of VANET [14]. [12] presented a hierarchical identity-based signature sharing scheme considering different types of communication hardware.

The parties involved in preserving privacy in the V2V semi-honest trust model is not suitable for provisioning safety-related application. To preserve the privacy of user data, in the literature, may researchers have adopted cryptography techniques. The cryptography technique relies on the keys for encryption and decryptions, where keys are symmetric or asymmetric in nature. To compute keys and distribute among parties, secure or third-party servers are considered, as seen in [12] and [13].

## 3. Proposed Secure Non-Cooperative Cognitive Division Multiple Access Model

This work presents a secure enhanced non-cooperative cognitive division multiple access (S−ENCCMA) model using a commutative RSA cryptography mechanism. This S−ENCCMA adopts the enhanced non-cooperative cognitive division multiple access (ENCCMA) [15] real-time MAC (Medium Access Control) communication protocol. To provision real-time access, the ENCCMA combines Time Division Multiple Access (TDMA), Frequency Division Multiple Access (FDMA), and the Cognitive Radio (CR) technique. The ENCCMA Medium Access Control protocol avoids signaling, which aids in enhancing the system efficiency. However, ENCCMA did not consider provisioning security for message authentication and user privacy. In the next subsection, the authors present our proposed security model.

### 3.1. Commutative RSA (CRSA) Model

Here, the authors present a secure and efficient implementation of the commutative RSA algorithm for message authentication among vehicles for the V2V environment. The notations used in the RSA commutative key are given in Table 1. In order to enable secure data communication among the corresponding vehicles in the V2V environment, a noble commutative RSA methodology that states that the order in which encryption is performed does not affect the result of the encryption, or the decryption can be done in a similar manner. In the majority of existing approaches, the public key cryptosystems employ a key exchange approach that ultimately causes the increase in computational overheads for key exchange; alternatively, at individual transceivers, the encryption and decryption are missed and thus somewhere the efficiency, as well as security, would be compromised. Therefore, the consideration of commutative RSA (CRSA) might be an optimum solution for accomplishing an efficient and most secure communication for multi-channel V2V vehicular ad-hoc smart infotainment applications. Here, the authors have proposed a secure CRSA cryptography algorithm, with the goal of enhancing the system performance for its lower memory occupancy and with higher throughput.

A secure communication model can be realized only when the message transmitted over the communication channel is protected and cannot be collided. To achieve the cryptography mechanism is generally considered. Therefore, the S−ENCCMA proposed here adopts the CRSA algorithm. The S−ENCCMA considers two prime param $A_{a}^{C}$ and $B_{L}^{C}$ initialized amongst all the vehicles of the region. Let ${ℛ}_{X}$ and ${ℛ}_{Y}$ represent the region member required to securely communicate over the secure channel. To compute the encryption keys and decryptions, key pairs of the CRSA algorithm—the propertord $L^{C}$ and $M^{C}$—are evaluated using the following

(1) $$L^{C}=\left[\left(A_{a}^{C}\right)\times \left(B_{b}^{C}\right)\right]$$

(2) $$M^{C}=\left[\left(A_{a}^{C}-1\right)\times \left(B_{b}^{C}-1\right)\right]$$

From the above expression, it can be seen that $L_{X}^{C}=L_{Y}^{C}$ and $M_{X}^{C}=M_{Y}^{C}$ for X and Y. The key pair for the encryption of X and B are signified as follows

(3) $$\left(L_{X}^{C},{ℰ}_{X}^{C}\right)\;\mathrm{and}\;\left(L_{Y}^{C},{ℰ}_{Y}^{C}\right)$$

The parameter ${ℰ}^{C}$ is obtained by arbitrarily choosing a parameter such that it is a co-prime of $M^{C}$, or in other terms,

(4) $$F_{G}\left({ℰ}^{C},M^{C}\right)=1$$

where $F_{G}\left(u,v\right)$ signifies the greatest common divisor function among u and v.

The key pair for the decryption of X and Y is depicted by $\left(L_{X}^{C},D_{X}^{C}\right)$ and $\left(L_{Y}^{C},D_{Y}^{C}\right)$ and the property $D^{C}$ is evaluated by using the following expression:

(5) $$D^{C}={\left({ℰ}^{C}\;\right)}^{-1}\left|L^{C}\right|.$$

Let $E_{U}$ denote the encrypted message U. The encryption process is expressed as follows

(6) $$E_{U}=V^{{ℰ}^{C}}\left|L^{C}\right|$$

The decryption process of CRSA on encrypted message Y is expressed as

(7) $$D_{V}=V^{D^{C}}\left|L^{C}\right|$$

### 3.2. Proof of CRSA Model

The commutative property of RSA adopted in the S−ENCCMA model can be proved if message U encrypted by X and then encrypted by Y provides the identical resultant to when the encryption is carried out by Y followed by encryption carried out by X, which can be stated as

(8) $$E^{Y}\left(E_{U}^{X}\right)\equiv E^{X}\left(E_{U}^{Y}\right)$$

(9) $$E^{Y}\left(U^{{ℰ}_{X}^{C}}\left|L_{X}^{C}\right|\right)\equiv E^{X}\left(U^{{ℰ}_{Y}^{C}}\left|L_{Y}^{C}\right|\right)$$

(10) $$U^{\left({ℰ}_{X}^{C}\times {ℰ}_{Y}^{C}\right)}\left|L_{X}^{C}\right|=U^{\left({ℰ}_{Y}^{C}\times {ℰ}_{X}^{C}\right)}\left|L_{Y}^{C}\right|$$

As $L_{X}^{C}=L_{Y}^{C}$ it can be said that

(11) $$U^{\left({ℰ}_{X}^{C}\times {ℰ}_{Y}^{C}\right)}\left|L_{X}^{C}\right|=U^{\left({ℰ}_{Y}^{C}\times {ℰ}_{X}^{C}\right)}\left|L_{X}^{C}\right|$$

Therefore,

(12) $$E^{Y}\left(E_{U}^{X}\right)\equiv E^{X}\left(E_{U}^{Y}\right)$$

**Figure 2:**
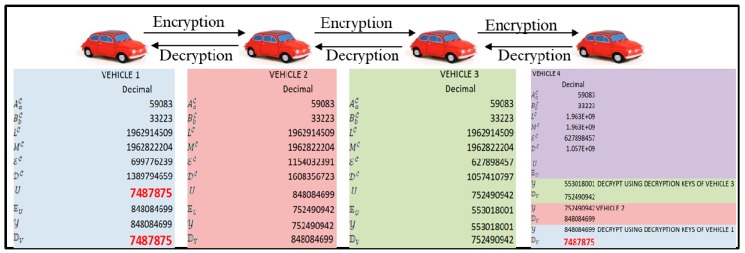
Secure enhanced non-cooperative cognitive division multiple access (S-ENCCMA) with Commutative Rivest–Shamir–Adleman (CRSA).

Each vehicle computes its public and private key using the proposed commutative RSA algorithm. Hop-based communication is adopted for data transmission among vehicles. Each vehicle encrypts the data using its own public key. The receiver performs decryption operation based on the number of times it is encrypted using its commutative keys of participating vehicles. The proposed model preserves the data and user’s privacy, and an intruder can be tracked using the user’s commutative keys. First, we established the key management where the key management center will distribute two prime numbers A and B to all VANETs, which are the same. Then, we will calculate L and M at each VANET node. Based on this, two vehicular nodes will compute the encryption and decryption keys. Second, we established the key exchange; now, once all the vehicles completed their encryption and decryption keys, they will inform the key management that it’s over. For instance (Figure 2), Vehicle 1 is the source and Vehicle 4 is the destination. Vehicle 1 has to send the data to Vehicle 4. So, what could happen is that the intermediate hops are Vehicle 2 and Vehicle 3. So, Vehicles 1, 2 and 3 will send their decryption key to the key management center, who will give these keys to only the destination vehicle, which is Vehicle 4. In secure data exchange, Vehicle 1 will then encrypt the data and send it to Vehicle 2. In normal RSA or normal Elliptic-curve cryptography (ECC), when you encrypt encrypted data again, data gets corrupted. So, on decryption, the data cannot be recovered (data lost). So, in our mechanism, the user does not need to decrypt the data; the user can just encrypt using his key and forward it. If there is an attacker that knows I’m Vehicle 3, now this attacker will not have the same encryption key. However, all the encryption keys are different from each vehicle, so the attacker will not be able to get the data. Even if the attacker gets the data, he will not be able to decrypt it because all the decryption keys are also different, and the attacker will not know how many times the data is encrypted. So, in this case, no one other than Vehicle 1 and Vehicle 4 will get to know the original data. That is the biggest advantage of securing complete VANET transmission. In addition, another advantage of this protocol that the intermediate nodes will not get to know the data content at all, because the data is encrypted multiple times and these multiple encryptions do not crop the data. In the normal RSA or ECC, every time you need to decrypt your data and then again re-encrypt it, because it does not support multiple encryptions and multiple decryptions.

In the next section, the simulation study proposed S−ENCCMA is evaluated.

## 4. Simulation Analysis and Result

The experiments are conducted on a Windows 10 operating system, 64−bit I−5 quad-core processor with 16 GB RAM and Dedicated 4 GB Nvidia CUDA GPU card. The SIMITS [13] simulator tool is used for experimental evaluation. The Proposed S−ENCCMA and existing ENCCMA [15] algorithm are written in C# object-oriented programing language using the Visual studio framework 4.5,2012. The S−ENCCMA and city, highway, and rural radio propagating environment model [Ours] is incorporated into the SIMITS tool [16,17,18,19,20]. Experiments are conducted to evaluate the performance of S−ENCCMA over ENCCMA in terms of throughput achieved, successful packet transmission and packet collision. The experiments are conducted considering different environments such as city, highway, and rural areas. The overall experiment is conducted to evaluate the overhead incurred in provisioning the proposed security model to ENCCMA protocol.

For modeling and simulating the environmental conditions of city, highway, and rural areas, we considered the parameters presented in [21]. These environmental parameters are as shown in Table 2 and have been obtained from a set of tests on IEEE 802.11p (5.9 GHz). This setup allows our model to achieve idealistic channel configuration. The simulation parameters considered for evaluation are shown in Table 3.

### 4.1. Throughput Performance

**Figure 3:**
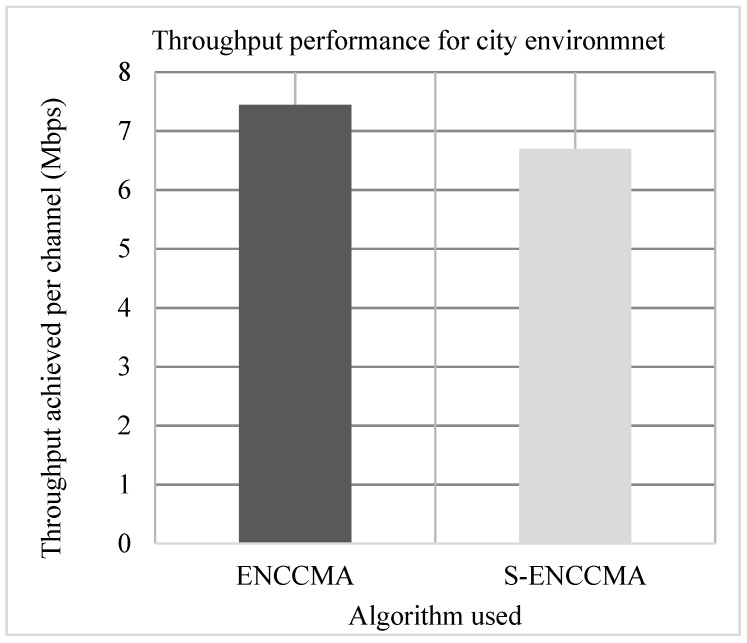
City environment throughput performance for varied vehicles.

**Figure 4:**
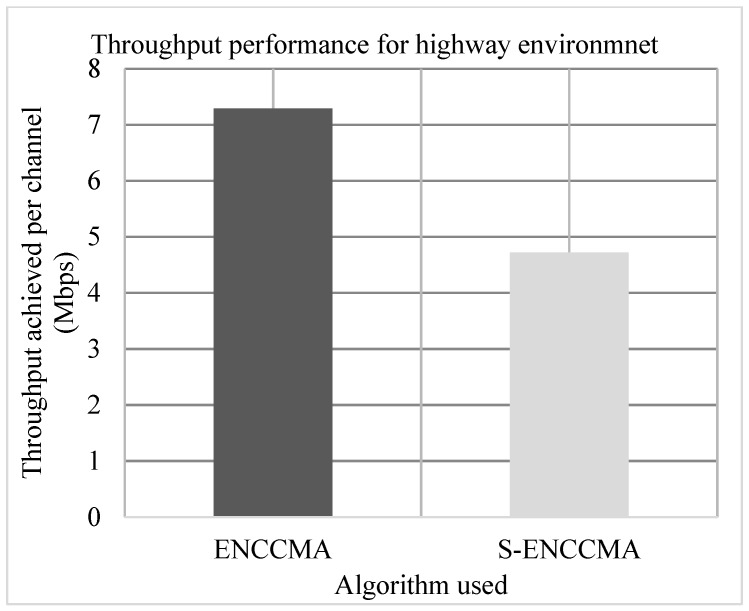
Highway environment throughput performance for varied vehicles.

**Figure 5:**
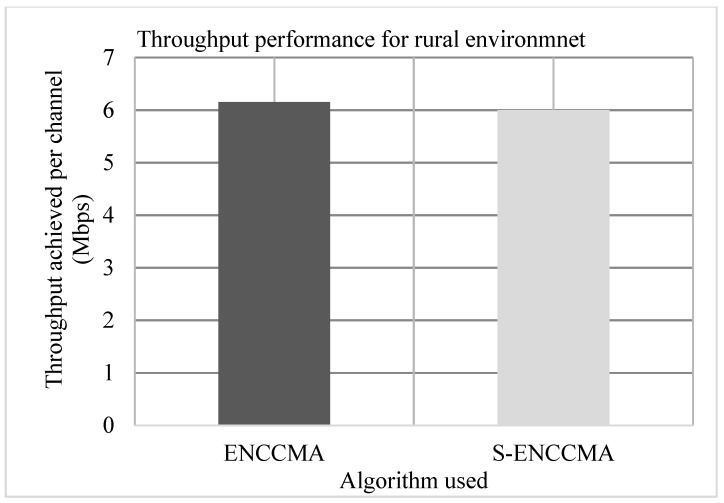
Rural environment throughput performance for varied vehicles.

Experiments are conducted to evaluate the throughput overhead incurred in provisioning security by the proposed security model considering 20 vehicles that are moving at a speed of 20 m/s. Figure 3, Figure 4 and Figure 5 show the throughput performance of the proposed S−ENCCMA with security and existing ENCCMA without security for city, highway and rural environments, respectively. The experiment outcomes show that ENCCMA and S−ENCCMA achieve an average throughput of 6.96 Mbps and 5.8 Mbps respectively considering varied environments. The overall result obtained shows that provisioning security to ENCCMA protocol incurs a throughput overhead of 15.91% considering varied environments.

### 4.2. Successful Packet Transmission Performance

**Figure 6:**
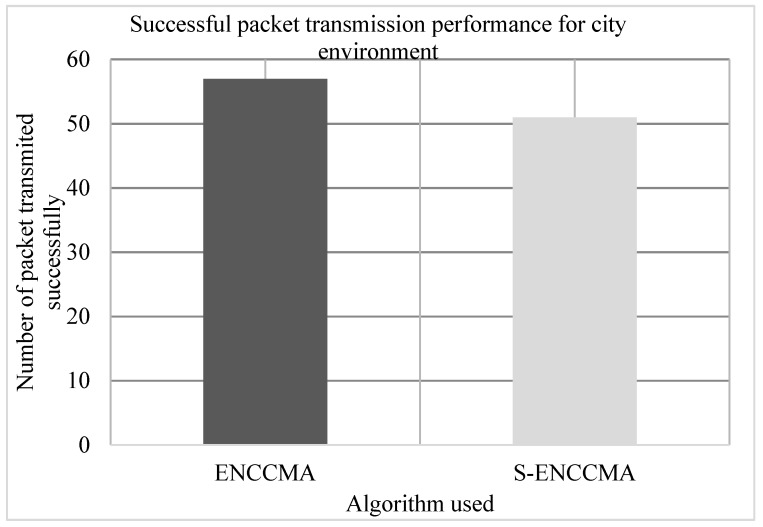
Successful packet transmission performance achieved for a city environment.

**Figure 7:**
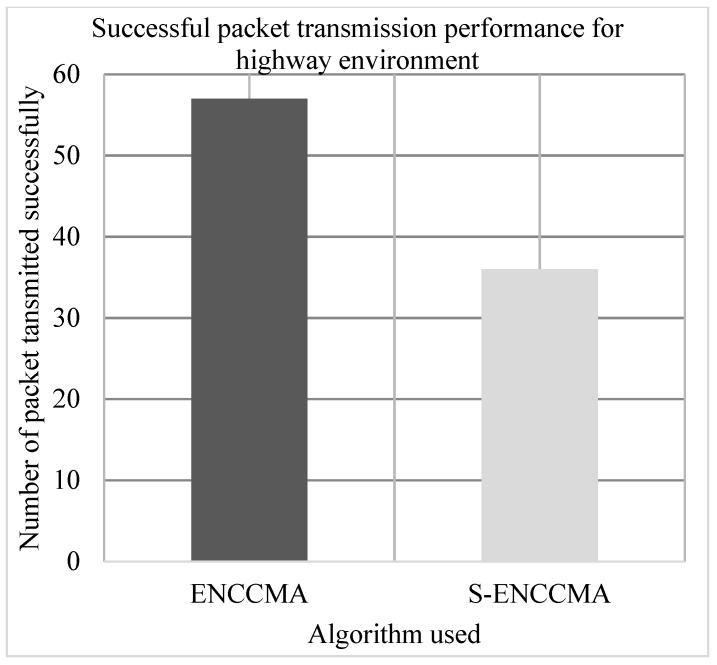
Successful packet transmission performance achieved for a highway environment.

**Figure 8:**
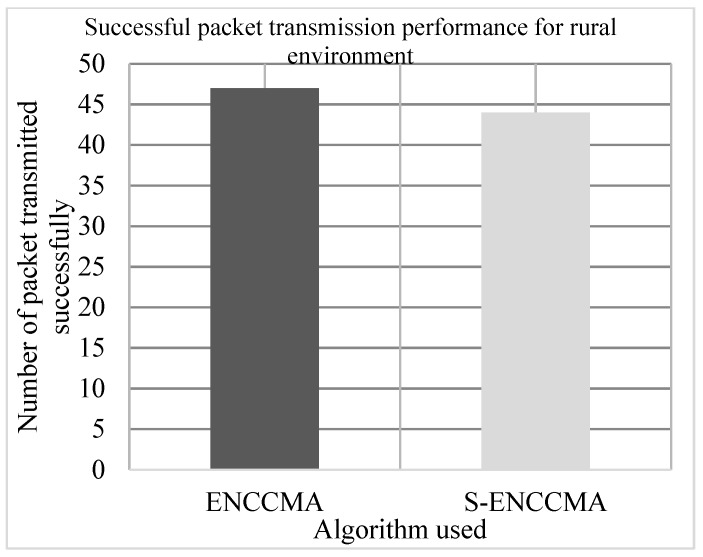
Successful packet transmission performance achieved for a rural environment.

The experiment is conducted to evaluate the successful packet transmission overhead incurred in provisioning security by the proposed security model. Figure 6, Figure 7 and Figure 8 show the throughput performance of the proposed S−ENCCMA with security and existing ENCCMA without security for city, highway and rural environments, respectively. The experiment outcomes show that the ENCCMA and S−ENCCMA achieve an average successful packet transmission of 53.66 Mbps and 43.66 Mbps respectively considering varied environments. The overall result obtained shows that provisioning security to ENCCMA protocol incurs a successful packet transmission overhead of 17.97% considering varied environments.

### 4.3. Collision Performance

**Figure 9:**
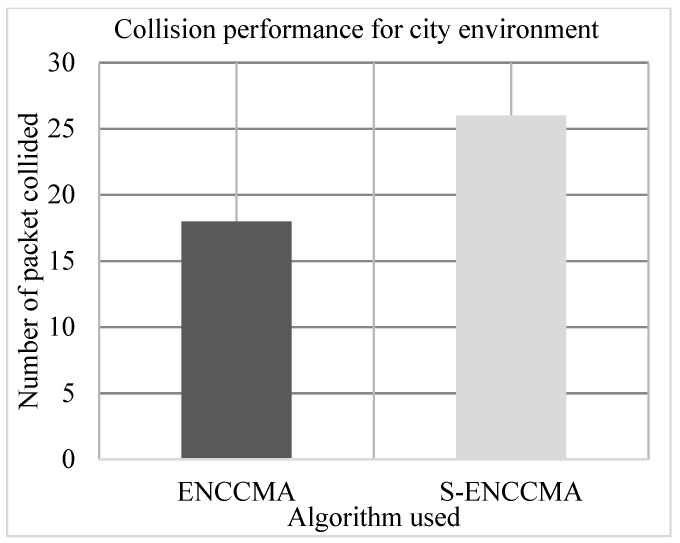
Collision performance achieved for a city environment.

**Figure 10:**
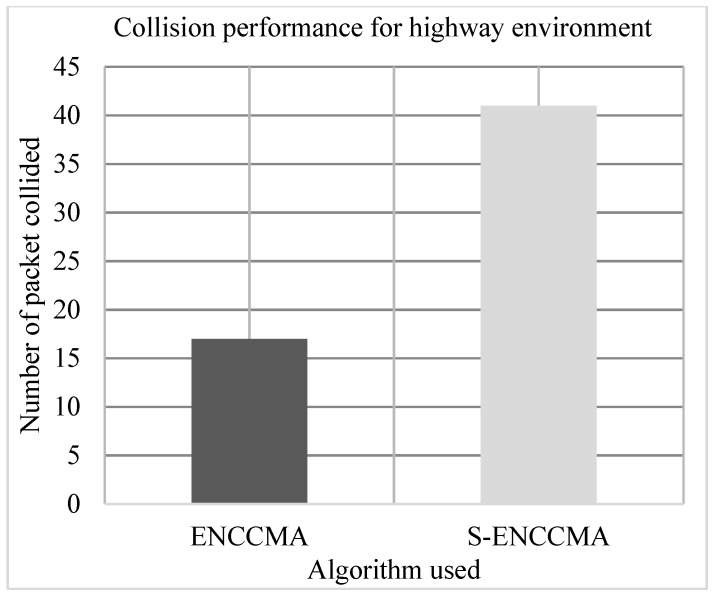
Collision performance achieved for a highway environment.

**Figure 11:**
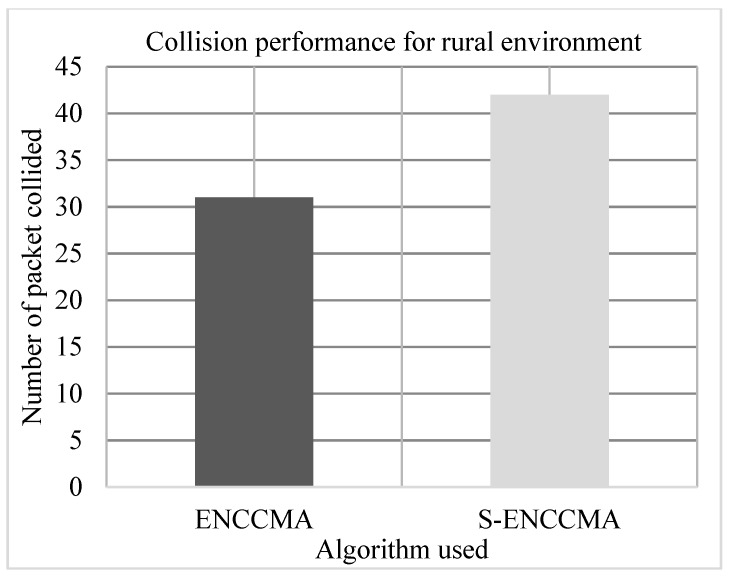
Collision performance achieved for a rural environment.

The experiment is conducted to evaluate the collision overhead incurred in provisioning security by the proposed security model. Figure 9, Figure 10 and Figure 11 show the collision performance of proposed S−ENCCMA with security and existing ENCCMA without security for city, highway, and rural environments, respectively. The experiment outcomes show that the ENCCMA and S−ENCCMA achieve an average collision of 22.0 Mbps and 36.33 Mbps respectively, considering varied environments. The overall result obtained shows that provisioning security to ENCCMA protocol incurs a collision overhead of about 38.91% considering varied environments.

**Table 4:** Comparison with state of-art-techniques.

	S-ENCCMA (Ours)	ENCCMA [15]	Mobile Slotted Aloha (MS-ALOHA) [22]	Slotted Period (SLOP) [23]	Earliest Deadline First based Carrier Sense Multiple Access (EDF-CSMA) [24]
Environmental model used	City, Highway, and Rural (CHR)	Freely flowing vehicles	Urban and highway	Intelligent driver	NA
Scheduling Algorithm Used	CRSA	ENCCMA (NCC-TDMA-FDMA)	MS-ALOHA	Wave-Slotted aloha	EDF-CSMA
Simulator used	SIMITS	SIMITS	VISSIM	YES (NA)	NS-3
MAC USED	802.11p MAC	802.11p MAC	802.11p MAC	802.11p MAC	802.11p MAC
Channel sharing available	Yes	Yes	No	No	No

### 4.4. Comparison with State of Technique

In Table 4, a comparison of S-ENCCMA with a state-of-art technique is showed. It shows that S-ENCCMA support distributes channel-sharing mechanisms for the V2V environment. Thus, S-ENCCMA aids in maximizing the system throughput with minimum collision overhead and hence enhancing system efficacy. The S−ENCCMA adopts the enhanced non-cooperative cognitive division multiple access (ENCCMA) [15] real-time MAC (Medium Access Control) communication protocol. To provision real-time access, the ENCCMA combines Time Division Multiple Access (TDMA), Frequency Division Multiple Access (FDMA), and Cognitive Radio (CR) techniques. The ENCCMA Medium Access Control (MAC) protocol avoids signaling, this aids in enhancing the system’s efficiency. However, ENCCMA did not consider provisioning security for message authentication and user privacy. In [23], they evaluated the packet delivery performance considering different environments. However, they did not consider varying the number of devices and vehicle mobility in their experiments. In [23,24], the authors conducted the experimental analysis considering varied mobility speeds to evaluate the collision performance. However, the performance evaluation under different environmental conditions was not considered. Our model considers performance evaluation considering throughput, collision and successful transmission considering a varied number of vehicles, varied speed, and varied environmental conditions. The overall survey shows the efficiency of our model over state-of-the-art techniques.

## 5. Discussion

A malicious user can sit in a parking lot and pick up communications from several miles away through digging up cables or gaining physical access to a router. Their own wireless signal can be created or interjected by a malicious user. So, it is very important that the communications for traffic monitoring be safe and secured. The accidental or malicious actions that can cause disruption can be prevented. Even though malicious attacks on traffic monitoring might seem far-fetched, they appear quite plausible if you examine the gray market devices people now buy to minimize travel time (such as infrared transmitters to redo traffic lights). To reduce a particular driver’s travel time or toward a particular roadway to increase revenue at a particular store, these new devices might manipulate the congestion index to divert traffic away from a road. The information quality of a competing traffic-monitoring service might be diluted by other service providers for four reasons, and a result, protecting the ITS communications network throughout the system design phase is crucial. First, the most effective phase at which to limit exposures is presented by system design. Second, the research can be done in a limited expenditure by considering security early, which is unlikely to be securable. Third, the possibility of attacks is ignored, which can cause incorrect conclusions about system robustness. Finally, to garner governmental approval and consumer acceptance, the security is crucial. VANET is very useful in recent times, as it can be applied to the safety application. As the various broadcast messages in the VANET are safety-related, it needs deep penetration and less delivery time. The security of these messages is also important; the message must be authorized and not leaked, as the owner of the vehicle has the right of privacy. Security has attracted less attention so far. VANET packets contain life-critical information; hence, it is necessary to make sure that these packets are not inserted or modified by the attacker. Likewise, the liability of drivers should also be established that they inform the traffic environment correctly and in time. These security problems are not similar to a general communication network. The size of the network, mobility, geographic relevancy, etc., makes the implementation difficult and distinct from other network security.

In VANET, each vehicle computes its public and private keys using the proposed commutative RSA algorithm. Hop-based communication is adopted for data transmission among vehicles. Each vehicle encrypts the data using its own public key. The receiver performs decryption operation based on the number of times it is encrypted using the commutative keys of participating vehicles. This work presents a secure enhanced non-cooperative cognitive division multiple access (S−ENCCMA) model using a commutative RSA cryptography mechanism. This S−ENCCMA adopts the enhanced non-cooperative cognitive division multiple access (ENCCMA) real-time MAC (Medium Access Control) communication protocol. To provision real-time access, the ENCCMA combines Time Division Multiple Access (TDMA), Frequency Division Multiple Access (FDMA), and Cognitive Radio (CR) techniques. The ENCCMA Medium Access Control protocol avoids signaling, which aids in enhancing system efficiency. However, ENCCMA did not consider provisioning security for message authentication and user privacy. Here, the authors present a secure and efficient implementation of the commutative RSA algorithm for message authentication among vehicles for the V2V environment. The notations used in the RSA commutative key are given in Table 1. In order to enable secure data communication among the corresponding vehicles in the V2V environment, a noble commutative RSA methodology that states that the order in which encryption is performed does not affect the result of the encryption or the decryption can be done in a similar manner. For modeling and simulating the environmental conditions of city, highway and rural areas, the authors considered the parameters presented in [20]. These environmental parameters are as shown in Table 2 and have been obtained from a set of tests on IEEE 802.11p. This setup allows our model to achieve an idealistic channel configuration. Experiments are conducted to evaluate the throughput, successful packet transmission, and collision overhead that are incurred in provisioning security by the proposed security model considering 20 vehicles that are moving at a speed of 20 m/s. The result shows provisioning security to ENCCMA protocol incurs a throughput overhead of 15.91%, a successful packet transmission overhead of 17.97%, and a collision overhead of 38.91% considering varied environments. The outcome shows that overhead incurred by S−ENCCMA over ENCCMA was negligible regarding the provision of the real-time security requirements of smart infotainment applications, which is experimentally proven.

## 6. Conclusions

Providing security for smart infotainment applications in VANET is most desired. As a result, it requires an efficient authentication mechanism that preserves the user’s privacy and security requirements of VANET. Many existing schemes to preserve privacy have adopted cryptography approaches. However, it incurs key management and communication overhead. To address this, this work presents a novel security scheme—namely, secure non-cooperative cognitive medium access S−ENCCMA. In the majority of existing approaches, the public key cryptosystems employ a key exchange approach that ultimately causes the increase in computational overheads for key exchange; alternatively, an individual transceiver requires encryption and decryption, and thus somewhere the efficiency, as well as security, would be compromised. Therefore, the consideration of commutative RSA (CRSA) might be an optimum solution for accomplishing an efficient and most secure communication for multi-channel V2V vehicular ad hoc smart infotainment applications. Here, we proposed secure RSA with a commutative key cryptography algorithm, with a goal of enhancing the system performance for its lower memory occupancy and with higher throughput. Here, each vehicle computes its public and private key using the proposed commutative RSA algorithm. Hop-based communication is adopted for data transmission among vehicles. Each vehicle encrypts the data using its own public key. The receiver performs a decryption operation based on the number of times it is encrypted using its commutative keys of participating vehicles. The experiment is conducted to evaluate the overhead incurred in provisioning security to ENCCMA. The S−ENCCMA protocol incurs an average throughput overhead of 15.91%, an average collision overhead of 38.91%, and an average success packet transmission overhead of 17.97% when security is provisioned to ENCCMA considering the different environmental conditions. The outcome shows that the overhead incurred by S-ENCCMA over ENCCMA was negligible regarding the provision of the real-time security requirements of smart infotainment applications, considering the varied environmental model. However, future work would consider a comparison between the performances of the proposed model with the performances of the existing models using more than 20 vehicles. A field study is needed to validate the results of this study with a real-world use case that develops a new MAC that further reduces the collision and improves the throughput and packet transmission efficiency. For this, we considering provisioning the proposed security design. 

## Figures and Tables

**Table 1:** Variable notation.

Notation	Meaning
$A_{a}^{C}$	p: Prime Number
$B_{b}^{C}$	q: Prime Number
$L^{C}$	n = p × q
$M^{C}$	Phi = (p − 1) × (q − 1): Enter totient number of (n) and φ(n).
${ℰ}^{C}$	e: Public Key
$D^{C}$	d: Secret Key
U	Data
$E_{U}$	Enc = Data^e mod(n)
Y	EncData
$D_{V}$	Dec = EncData^d mod(n)

**Table 2:** Channel parameters for different environments used for simulation [21].

Environment	City	Highway	Rural
Path loss	1.61	1.85	1.79
Shadowing deviation	3.4	3.2	3.3

**Table 3:** Parameters. MAC: Medium Access Control.

Parameters	Value
Network	30 km × 30 km
MAC	ENCCMA and S−ENCCMA
Modulation scheme	64-QAM
Mobility of vehicles	20 cycle per frame
Bandwidth	27 Mbps
Frequency channels	7
Vehicles	20
Coding rate	0.75
Message size	75 bytes
Time slots	8 μs
Environment	Rural, City & Highway
